# Novel Intrinsic Ignition Method Measuring Local-Global Integration Characterizes Wakefulness and Deep Sleep

**DOI:** 10.1523/ENEURO.0106-17.2017

**Published:** 2017-09-22

**Authors:** Gustavo Deco, Enzo Tagliazucchi, Helmut Laufs, Ana Sanjuán, Morten L. Kringelbach

**Affiliations:** 1 Center for Brain and Cognition, Computational Neuroscience Group, Department of Information and Communication Technologies, Universitat Pompeu Fabra, Barcelona 08018, Spain; 2 Institució Catalana de la Recerca i Estudis Avançats (ICREA), Universitat Pompeu Fabra, Barcelona 08010, Spain; 3 Institute for Medical Psychology, Christian Albrechts University Kiel, Kiel 24105, Germany; 4 Department of Neurology and Brain Imaging Center, Goethe University Frankfurt am Main, Frankfurt am Main 60528, Germany; 5 Department of Neurology, Christian Albrechts University Kiel, Kiel 24104, Germany; 6 Department of Psychiatry, University of Oxford, Oxford OX3 7JX, United Kingdom; 7 Center for Music in the Brain, Department of Clinical Medicine, Aarhus University, Denmark; 8 Institut d’études Avancées de Paris, France

**Keywords:** whole-brain modeling, neuroimaging, ignition, metastability, integration

## Abstract

A precise definition of a brain state has proven elusive. Here, we introduce the novel local-global concept of intrinsic ignition characterizing the dynamical complexity of different brain states. Naturally occurring intrinsic ignition events reflect the capability of a given brain area to propagate neuronal activity to other regions, giving rise to different levels of integration. The ignitory capability of brain regions is computed by the elicited level of integration for each intrinsic ignition event in each brain region, averaged over all events. This intrinsic ignition method is shown to clearly distinguish human neuroimaging data of two fundamental brain states (wakefulness and deep sleep). Importantly, whole-brain computational modelling of this data shows that at the optimal working point is found where there is maximal variability of the intrinsic ignition across brain regions. Thus, combining whole brain models with intrinsic ignition can provide novel insights into underlying mechanisms of brain states.

## Significance Statement

We introduce a novel intrinsic ignition method for characterizing the dynamical complexity of different brain states. Naturally occurring intrinsic ignition events reflect the capability of a given brain area to propagate neuronal activity to other regions, giving rise to different levels of integration. We show that the intrinsic ignition method can clearly distinguish human neuroimaging data of two fundamental brain states (wakefulness and deep sleep). Furthermore, we use whole-brain modeling to show that the optimal working point is found where there is maximal variability of intrinsic ignition across brain regions.

## Introduction

At first glance, defining a brain state may seem simple, yet a useful definition has proven elusive (Friston et al., 2003; Deco et al., 2009). There could be many reasons for this failure but the main reason is likely to come from the realisation that whole-brain dynamics are much more complex than previously thought, and that for example traditional attractor states do not adequately describe them (Amit, 1989). Here, we propose that a given brain state could be defined by its dynamical complexity, understood as the broadness of communication.

Indeed, the dynamical complexity of the underlying brain state must arise from the interplay between anatomy and functional dynamics (Ghosh et al., 2008; Deco et al., 2011). For a given brain state, a balance has to be found between the integration and segregation of information (Deco et al., 2015). The dynamical repertoire of a brain state depends on the underlying anatomic structural connectivity (SC) and local dynamics (Deco and Kringelbach, 2016), and a number of different methods have tried to describe the spatiotemporal unfolding of activity (Allen et al., 2014; Hansen et al., 2015). These methods are able to describe the evolution of global whole-brain activity but they are less good at describing the interaction of how activity in a local region shapes global activity, i.e., at describing how the broadness of communication is elicited and distributed.

Here, we propose a conceptual framework for studying the intrinsic ignition of brain activity across time and space, i.e., the diversity of computation in space and time. In other words, intrinsic ignition refers to the capability of a given brain area to propagate feed-forward and recurrent neuronal activity to other regions, importantly in the absence of extrinsic perturbations (natural or artificial stimulations). This novel concept allows the study not only of the propagation of brain activity but also of the underlying fluctuations and their functional network consequences, i.e., the integration of information over the whole-brain network (Deco et al., 2015). Intrinsic ignition quantifies the capability of a given local brain region to propagate neuronal activity to other regions in the global whole-brain network.

Furthermore, by defining the mean and variability of the ignition-driven propagation of activity across regions, we can characterize the hierarchical organization of the whole-brain network, that is provide a measure of the ability of different regions to ignite the integration of information. Ranking brain regions by their intrinsic ignition provides a fingerprint of the hierarchical connectivity or dynamical processing hierarchy of a given brain state within the structural connectome. There are many possibilities, from weak to strong hierarchical processing in which the different brain regions are playing different roles dependent on shape and form of the brain state fingerprint.

This novel concept of intrinsic ignition is complementary to the approach taken by Dehaene and colleagues (Moutard et al., 2015), where they define ignition as the rapid and sometimes sustained activity elicited after stimulation by external stimuli. Their concept could thus be thought of as extrinsic ignition. Both modes of intrinsic and extrinsic ignition can emerge from the same underlying connectome as “two dynamic faces” of the strong recurrent loops built by brain networks. Indeed, the dense lateral intra- and interareal connections that characterize brain networks make possible the emergence of a reverberatory dynamics when the level of excitation exceeds the level of inhibition which can be propagated globally across the brain. This imbalance between excitation and inhibition could appear spontaneously in the resting state (intrinsic ignition) or rapidly induced (extrinsic ignition) by the action of stimulation by extrinsic sensory stimuli, explaining in this way both modes. Nevertheless, this concept of extrinsic ignition does not explain how an intrinsic local activity event in a given brain state (e.g., wakefulness, sleep) is eliciting a propagation of activity across the whole-brain network.

Here, we apply the novel concept of intrinsic ignition to characterize the dynamical complexity and broadness of communication in a unique human neuroimaging dataset measuring the naturally occurring brain states of sleep and wakefulness. Furthermore, we construct a whole-brain model of these brain states to explore the causal link between intrinsic ignition and the dynamical regime, to deepen our understanding of the dynamical complexity underlying brain states.

## Materials and Methods

### Experimental design

The objectives of the study are to introduce and demonstrate the usefulness of our novel intrinsic ignition method for characterizing the fundamental brain states. We designed the study to use this method to characterize neuroimaging data of human participants in wakefulness and sleep.

### Participants

A total of 63 young healthy consecutive subjects with data of sufficient quality were included in the study (written informed consent, approval by the local ethics committee, participants were reimbursed for their participation). Subjects were scanned with simultaneous EEG-fMRI in the evening after following a regular sleeping schedule. Eight subjects did not fall asleep inside the scanner and were excluded from the study, resulting in a group of five subjects who reached at least N1 sleep (36 females, mean ± SD age of 23.4 ± 3.3 years). Mean (±SD) durations of contiguous sleep epochs for these participants were 10.29 ± 9.45 min for wakefulness, 5.75 ± 4.84 min for N1, 6.14 ± 3.77 min for N2, and 11.67 ± 8.66 min for N3. In this article, we only considered the 18 participants that went through all three sleep stages, and we only considered the wakefulness and deep sleep (N3) conditions.

### fMRI and EEG acquisition and processing

EEG via a cap (modified BrainCapMR, Easycap) was recorded continuously during fMRI acquisition (1505 volumes of T2*-weighted echo planar images, TR/TE = 2080 ms/30 ms, matrix 64 × 64, voxel size 3 × 3 × 2 mm^3^, distance factor 50%; FOV 192 mm^2^) at 3T (Siemens Trio). An optimized polysomnographic setting was employed [chin and tibial EMG, ECG, EOG recorded bipolarly (sampling rate 5 kHz, low pass filter 1 kHz) with 30 EEG channels recorded with FCz as the reference (sampling rate 5 kHz, low pass filter 250 Hz); pulse oxymetry and respiration were recorded via sensors from the Trio (sampling rate 50 Hz)] and MR scanner compatible devices (BrainAmp MR+, BrainAmpExG; Brain Products), facilitating sleep scoring during fMRI acquisition (AASM, 2007; Jahnke et al., 2012). MRI and pulse artifact correction were performed based on the average artifact subtraction (AAS) method (Allen et al., 1998) as implemented in Vision Analyzer2 (Brain Products) followed by objective (CBC parameters, Vision Analyzer) ICA-based rejection of residual artifact-laden components after AAS resulting in EEG with a sampling rate of 250 Hz (Jahnke et al., 2012). EEG artifacts due to motion were detected and eliminated using an ICA procedure implemented in Vision Analyzer2. Sleep stages were scored manually by an expert according to the AASM criteria (AASM, 2007).

### fMRI preprocessing

Using Statistical Parametric Mapping (SPM8, www.fil.ion.ucl.ac.uk/spm) echo planar imaging (EPI) data were realigned, normalized (MNI space), and spatially smoothed (Gaussian kernel, 8-mm^3^ full width at half maximum). Data were resampled to 4 × 4 × 4 mm resolution to facilitate removal of noise and motion regressors. Note that resampling introduces averaging of blood oxygen level-dependent (BOLD) signals, which are nevertheless finally averaged over cortical and subcortical regions of interest to construct functional networks. Cardiac, respiratory (both estimated using the RETROICOR method; Glover et al., 2000), and motion-induced noise were regressed out. Data were bandpass filtered in the range 0.01-0.1 Hz (Cordes et al., 2001) using a sixth order Butterworth filter.

We used tools from FSL to extract and average the time courses from all voxels within each cluster in the automated anatomic labeling (AAL)90 atlas (i.e., the AAL atlas using cortical and subcortical but not cerebellar regions; Tzourio-Mazoyer et al., 2002) which were then used to constrain the global coupling of the Hopf model. The group functional connectivity (FC) matrix was averaged over the participants, using Matlab (The MathWorks) to compute the pairwise Pearson correlation between all 90 regions, applying Fisher’s transform to the *r* values to get the *z* values for the final 90 × 90 FC_fMRI matrix.

### Diffusion tensor imaging (DTI) acquisition and processing

The Hopf whole-brain model is constrained using the normal structural connectome obtained using DTI in 16 healthy right-handed participants (11 men and five women, mean age: 24.75 ± 2.54) who were recruited through the online recruitment system at Author University. Data were collected at Aarhus University, Denmark. Participants with psychiatric or neurologic disorders (or a history thereof) were excluded from participation in this study. The MRI data (structural MRI, DTI) were collected in one session on a 3T Siemens Skyra scanner at Aarhus University, Denmark. The parameters for the structural MRI T1 scan were as follows: voxel size of 1 mm^3^; reconstructed matrix size 256 × 256; echo time (TE) of 3.8 ms and repetition time (TR) of 2300 ms.

The DTI data were collected using TR = 9000 ms, TE = 84 ms, flip angle = 90°, reconstructed matrix size of 106 × 106, voxel size of 1.98 × 1.98 mm with slice thickness of 2 mm and a bandwidth of 1745 Hz/Px. Furthermore, the data were collected with 62 optimal nonlinear diffusion gradient directions at b = 1500 s/mm^2^. Approximately one nondiffusion weighted image (DWI; b = 0) per 10 diffusion-weighted images was acquired. Additionally, the DTI images were collected with different phase encoding directions. One set was collected using anterior to posterior phase encoding direction and the second acquisition was performed in the opposite direction. For the parcellation, we used the AAL template to parcellate the entire brain into 90 regions (76 cortical regions, adding 14 subcortical regions, AAL90). The parcellation consists of regions distributed in each hemisphere (Tzourio-Mazoyer et al., 2002). The linear registration tool from the FSL toolbox (www.fmrib.ox.ac.uk/fsl, FMRIB; Jenkinson et al., 2002) was used to coregister the EPI image to the T1-weighted structural image. The T1-weighted image was coregistered to the T1 template of ICBM152 in MNI space (Collins et al., 1994). The resulting transformations were concatenated and inversed and further applied to warp the AAL template (Tzourio-Mazoyer et al., 2002) from MNI space to the EPI native space, where interpolation using nearest-neighbor method ensured that the discrete labeling values were preserved. Thus the brain parcellations were conducted in each individual’s native space. We generated the SC maps for each participant using the DTI data acquired. We processed the two datasets acquired (each with different phase encoding to optimize signal in difficult regions). The construction of these SC maps or structural brain networks consisted of a three-step process. First, the regions of the whole-brain network were defined using the AAL template as used in the functional MRI data. Second, the connections between nodes in the whole-brain network (i.e., edges) were estimated using probabilistic tractography. Third, data were averaged across participants. Similar to the functional data, we applied the AAL90 template using the FLIRT tool from the FSL toolbox (www.fmrib.ox.ac.uk/fsl, FMRIB) to coregister the b0 image in diffusion MRI space to the T1-weighted structural image and then to the T1 template of ICBM152 in MNI space (Collins et al., 1994). The two transformation matrices from these coregistration steps were concatenated and inversed to subsequently be applied to warp the AAL templates (Tzourio-Mazoyer et al., 2002) from MNI space to the diffusion MRI native space.

### Hopf whole-brain computational model

The Hopf whole-brain computational model consists of 90 anatomically interconnected brain areas (cortical and subcortical nodes) defined according the AAL90 parcellation (see section on *Diffusion tensor imaging acquisition and processing*). More specifically, the underlying empirical anatomic SC matrix *C_ij_* couple the local dynamics of each brain area to emulate the empirically observed functional resting brain dynamics. In this way, the Hopf model explicitly link structure (anatomy) and function (BOLD signal). As described in the literature (Kringelbach et al., 2015; Deco and Kringelbach, 2016; Deco et al., 2017b), the whole-brain dynamics are given by the following set of coupled equations:

(1) $$\frac{dx_{j}}{dt}=\left[a_{j}-x_{j}^{2}-y_{j}^{2}\right]x_{j}-\omega_{j}y_{j}+G\sum_{i}C_{ij}\left(x_{i}-x_{j}\right)\beta \eta_{j}\left(t\right)$$

(2) $$\frac{dy_{j}}{dt}=\left[a_{j}-x_{j}^{2}-y_{j}^{2}\right]y_{j}+\omega_{j}x_{j}+G\sum_{i}C_{ij}\left(y_{i}-y_{j}\right)\beta \eta_{j}\left(t\right)$$

Where *η_j_* is additive Gaussian noise with standard deviation *β* = 0.02. The anatomic structural matrix *C_ij_*, coupling the local dynamics of nodes *i* and *j*, expresses the density of fibers between those brain areas as derived from DTI based tractography (which is here scaled to a maximum value of 0.2). The local dynamics of each individual brain area is described by a Landau-Stuart Oscillator which corresponds to the normal form of a supercritical Hopf bifurcation. The normal form of a Hopf bifurcation is the canonical model for studying the transition from noisy to oscillatory dynamics (Kuznetsov, 1998). Previous research has shown the usefulness, richness, and generality of this type of model for describing EEG dynamics at the local node level (Freyer et al., 2011, 2012). This normal form allowed us to fit the model to neuroimaging data over time, i.e., not only by fitting the grand average FC but also by fitting the temporal structure of the fluctuations, FC dynamics (Hansen et al., 2015).

The intrinsic frequency ω_i_ of each node of the network is in the 0.04–0.07 Hz band (*i* = 1, …,n). The averaged peak frequency of the narrowband BOLD signals of each brain region defines the intrinsic frequencies. The simulated BOLD signals were bandpass filtered within the narrowband 0.04–0.07 Hz, which is the frequency range that mapped to the gray matter has been shown to be more reliable and functionally relevant than other frequency bands in describing the resting state networks.

This normal form has a supercritical bifurcation at *a_j_ = 0*, so that for *a_j_ > 0* there exists a stable limit cycle oscillation with frequency *f_j_ = ω_j_/2π* and for *a_j_ < 0* the local dynamics has a stable fixed point at *z_j_ = 0* (which due to the additive Gaussian term corresponds to a low activity noisy state). Here, we take *a_j_ = 0* for all nodes, such that the local dynamics is described as a perfect mixture between noise and oscillations.

The variables *x_j_* emulate the BOLD signal of each node *j*. The parameter G denotes the global coupling corresponding to a factor scaling all synaptic conductivity connections. The global coupling parameter *G* is the control parameter with which we study the optimal dynamical working region where the simulations maximally fit the empirical data. More concrete, we study exhaustively how *G* is fitting the empirical FC matrix and the global coherence level. We fit the simulated FC matrices of the model (averaged Fisher's z-transformed over all sessions) to the empirical data (averaged Fisher’s z-transformed over all sessions) by maximizing the Pearson correlation coefficient between the elements of the upper triangular part of both empirical and simulated FC matrices. In addition, the global coherence is fitted by minimizing the absolute value of the difference between the empirical and simulated averaged global levels of synchronization between the different nodes across time (Wildie and Shanahan, 2012). We measure the averaged synchronization using the Kuramoto order parameter. The Kuramoto order parameter measures the global level of synchronization of the *n* oscillating signals. Under complete independence, the n phases are uniformly distributed and thus *R* is nearly zero, whereas *R* = 1 if all phases are equal (full synchronization). The mathematical expression of the Kuramoto order parameter is given by:

(3) $$R(t)=\left|\sum_{k=1}^{n}e^{i\varphi_{k}(t)}\right|/n$$

where *φ_k_(t)* is the instantaneous phase of each narrowband BOLD signal at node *k*. The instantaneous phase *φ_k_(t)* of each narrowband signal was computed using the Hilbert transform. The Hilbert transform yields the associated analytical signals. The analytic signal represents a narrowband signal, *s(t)*, in the time domain as a rotating vector with an instantaneous phase, *φ(t)*, and an instantaneous amplitude, *A(t)*, i.e., s(t)=A(t)cos(φ(t)). The phase and the amplitude are given by the argument and the modulus, respectively, of the complex signal *z(t)*, given by z(t)=s(t)+i.H[s(t)], where i is the imaginary unit and *H*[*s*(*t*)] is the Hilbert transform of *s(t)*.

### Ignition-driven mean integration (IDMI)

**Figure 1.**
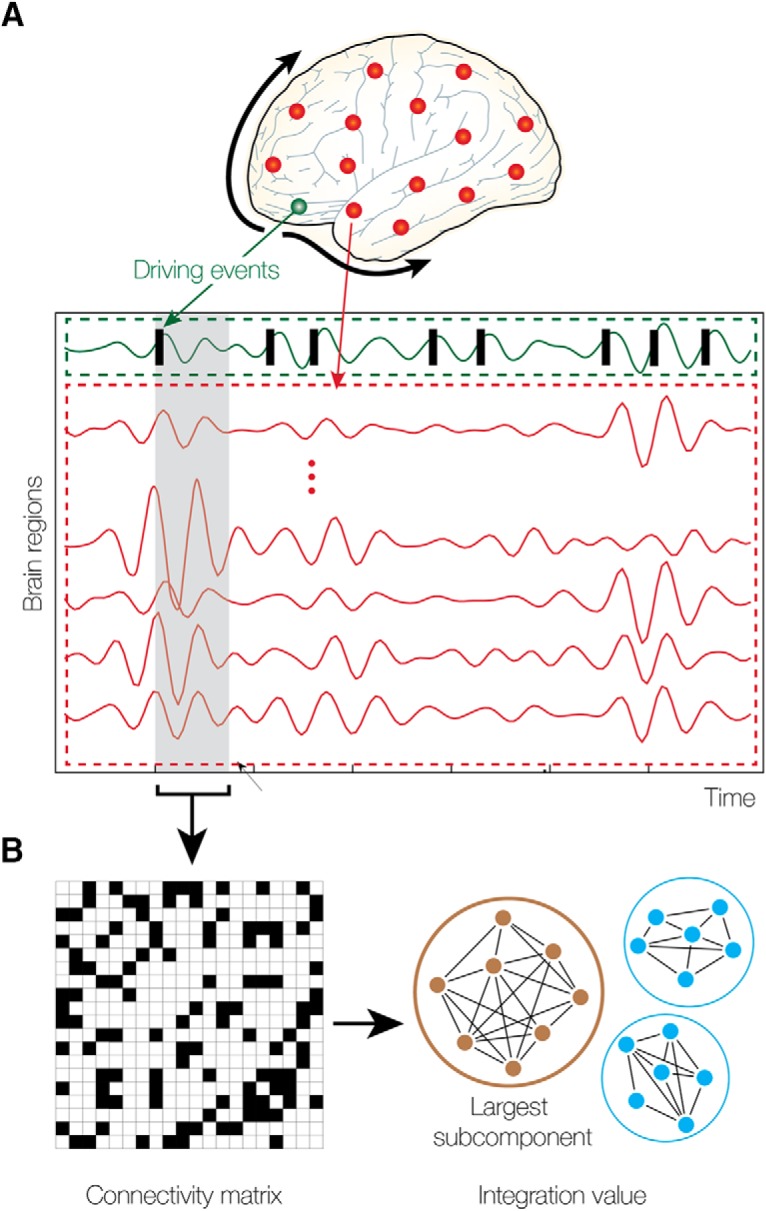
Measuring intrinsic ignition. ***A***, The activity of a region in the network can be measured using BOLD neuroimaging signals where a threshold method can be used to define events as those crossing a threshold from below (see green line and Materials and Methods). For each driving event, we measure the activity in the rest of the network (in stippled red area) in the gray time window. ***B***, This corresponds to a binary phase lock matrix over the time window (left panel). In this matrix, we can compute the integration by finding the largest subcomponent (computing the area under the curve for all integration values for all thresholds). This integration is a measure of the global integration, i.e., the broadness of communication across the network (Deco et al., 2015). This can be repeated for each of the driving events, producing a mean of the intrinsic ignition for each network region, which we call *Ignition-Driven Mean Integration* (IDMI).

Within our novel intrinsic ignition framework the definition of an intrinsic ignition event is as follows: An intrinsic ignition event for a given brain region is defined by binarizing the transformed functional time series (BOLD fMRI) into z-scores *z_i_*(*t*) and imposing a threshold θ such that the binary sequence *σ_i_*(*t*) = 1 if *z_i_*(*t*) > θ, and is crossing the threshold from below, and *σ_i_*(*t*) = 0 otherwise. This procedure is described in details by Tagliazucchi et al. (2012a) and in Figure 1*A*.

We investigate how a global measure of dynamical complexity, namely the integration, evolves in a window of time when triggered by the events of that given single brain region (Fig. 1). More specifically, for a given brain region, we average across the events a measure of the integration elicited at time *t* relative to the events. Finally, we define the IDMI of a given brain area as the averaged elicited integration during a time window of four TRs. The selection of this window width was determined by the time that takes the integration to return to basal values. We repeated this procedure for all brain regions in the AAL90 parcellation.

We compute the integration using the phase space of the signals. We call this measure phase-based integration. We first filter the fMRI signals in the range of 0.04-0.07 Hz as explained above and extract via the Hilbert transform the phases. For each time point we calculate the phase lock matrix describing for each time point the state of pair-wise phase synchronization between regions *j* and *k* as:

(4) $$P_{jk}(t)=e^{-3|\varphi_{j}(t)-\varphi_{k}(t)|}$$

where $\varphi_{j}(t)$ is the extracted phase of brain area *j* at time *t*. Then we compute the level of integration associated with that coherence configuration at time *t.* The concept of integration can be defined using the length of the largest connected component in the phase lock matrix $P_{jk}(t)$. More specifically, for a given absolute threshold θ between 0 and 1 (scanning the whole range), the symmetric phase lock matrix $P_{jk}(t)$ can be binarized (0 if |P*_jk_*|<θ, 1 otherwise). From this symmetric phase lock matrix, a value of integration is computed as the largest component given by the length of the connected component of the undirected graph defined by the binarized matrix considered as an adjacency matrix. This is the largest subgraph in which any two vertices are connected to each other by paths, and which connects to no additional vertices in the supergraph.

### Ignition-driven variability

To characterize the degree of hierarchical organization of the brain, we calculate ignition-driven variability by computing the standard deviation of the IDMI across nodes.

#### Surrogate analysis

To assess the validity of our ignition measures, we test whether the ignition values obtained from the real data are significantly higher than those obtained from reshuffled, randomized data. To create the random data, we just permuted (random permutation) the time series phases in each time point and then compute the ignition values associated for our spontaneous events. We repeat this procedure 50 times, and then compare the average of these values to the ignition values obtained on the real data.

#### Permutation tests

We tested whether there were significant differences between conditions using a Monte Carlo permutation test. To this end, for each pair of conditions, we randomly shuffle the labels between the conditions to create two new simulated conditions, repeat this procedure in an iterative way (number iterations, 10,000) and asses how many times the difference between the simulated conditions is higher than the difference between the condition to be compared. In conclusion, we compute the *p* value of the null hypothesis that the two random distributions show higher difference than the conditions to be compared. We used this approach to compare the ignition values for the real versus randomized data and also to compare the ignition values across real conditions.

## Results

### Intrinsic ignition

We investigated a unique human neuroimaging dataset with two fundamentally different brain states, namely wakefulness and deep sleep (N3 stage of sleep). First, we used the novel intrinsic ignition method to characterize the broadness of communication in the two states. In the Materials and Methods section and in Figure 1, we describe the precise algorithm for computing intrinsic ignition. Essentially, we compute the intrinsic ignition for a given region by computing the integration in a time window following an event in this region. We then compute the average across events and the variability across nodes. The profile of the mean and variability of intrinsic ignition triggered integration as a function of the brain region characterize the dynamical complexity underlying a certain brain state.

Briefly, an intrinsic ignition event is defined as a binary signal resulting from the transformed functional time series (BOLD fMRI) into z-scores *z_i_*(*t*) and imposing a threshold θ such that the binary sequence *σ_i_*(*t*) =1 if *z_i_*(*t*)> θ, and is crossing the threshold from below, and σ_i_(t) = 0 otherwise (see Materials and Methods; Tagliazucchi et al., 2012a, and their Fig. 1*A*). Figure 1 shows a cartoon of this procedure. As can be seen, if the top signal (in red) refers to the specific brain region whose ignition capability we are analyzing, an event is the point where the signal crosses the threshold from below.

**Figure 2.**
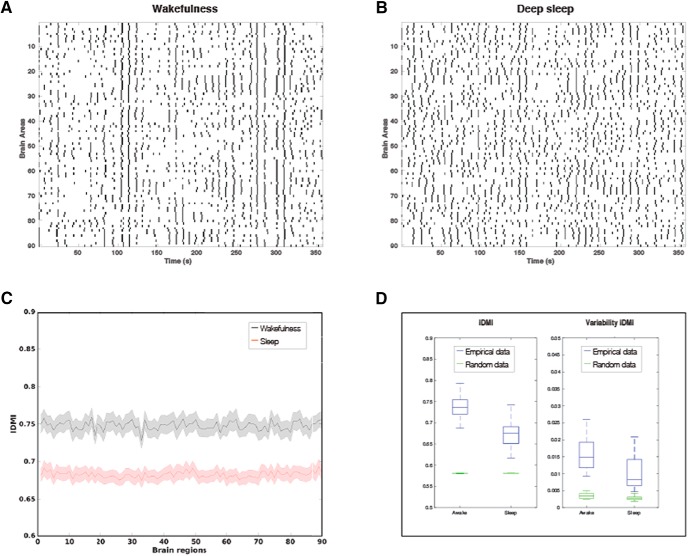
The *Ignition-Driven Mean Integration* (IDMI) for wakefulness and deep sleep. ***A***, Example of intrinsic ignition events for a time window in one wakeful participant. ***B***, Example of intrinsic ignition events for a participant in deep sleep across all brain regions. ***C***, We plot the IDMI in wakefulness (top curve) and deep sleep (bottom curve) for all of the 90 AAL brain regions across all participants. They are clearly separated. ***D***, Box plot shows that the IDMI across participants are significantly different between wakefulness and sleep in terms of both mean (left blue graphs, IDMI) and variability (right blue graphs, variability of IDMI) across brain regions. In addition, we show that computing IDMI on surrogate (in green), reshuffled data are significantly lower, showing the specificity of this novel measure.

Figure 2*A*,*B* shows for a particular subject the events for all BOLD signals under two different brain states, namely wakefulness and sleep (N3 stage). The *x*-axis represents the time whereas the *y*-axis represents the different brain areas. Here, we parcellate the brain using the AAL90 (automatic anatomy labeling, including all cortical and subcortical areas; Tzourio-Mazoyer et al., 2002). Each single black vertical bar refers to an event for the corresponding brain area.

We compute the IDMI across events of a given brain area as the averaged elicited integration during a time window of four TRs. Figure 2*C* plots the IDMI based on the phase lock matrix (see phase-based integration definition in Materials and Methods). Figure 2, bottom left panel, plots the IDMI under two different conditions, namely wakefulness and sleep, for all brain areas. It is very clear from the figure (and explicitly analyzed in Fig. 2*D*), that the mean value of the IDMI across brain areas is significantly different for both conditions (*p* < 0.0001, with the data in blue and the surrogate data in green).

We also compute the variability of IDMI across brain regions by computing the standard deviation, which is also significantly different for both conditions (*p* < 0.005). It is important to remark that the variability of IDMI is a good marker for the hierarchy of computation (Deco and Kringelbach, 2017). If the variability is small, this suggests that the brain organization is less hierarchical while larger variability suggests more hierarchical.

Finally, to test the validity of the ignition method, we computed the ignition values on reshuffled, randomized data. Our results show that the ignition values obtained from the randomized data are significantly smaller than the ignition values obtained from the real data (*p* < 0.0001 for both the IDMI and variability of IDMI; Fig. 2*D*, green)

### Exploration using the Hopf whole-brain computational model

**Figure 3.**
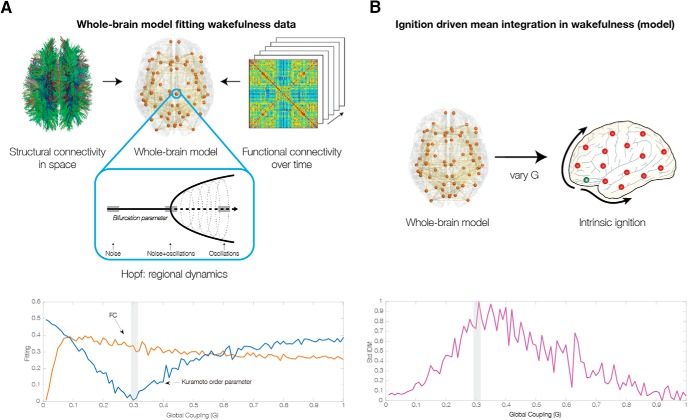
Relating intrinsic ignition to causal whole-brain computational modeling. ***A***, First, we fit the whole-brain Hopf model to the neuroimaging wakefulness data using functional and structural neuroimaging data. We plot the fit of the model in terms of grand average functional connectivity (FC) and synchronization as a function of the global coupling parameter (G), with the gray bar indicating the optimal coupling for the model. ***B***, We compute the intrinsic *Ignition-Driven Mean Integration* (IDMI) for each of the global coupling values of the model. As can be seen at the optimal coupling point (marked with the gray bar), we also find a maximal value of the standard deviation of IDMI across brain regions. This suggests that resting wakefulness contains maximal hierarchical organization.

To further explore the causal link between intrinsic ignition and the dynamical regime, we construct a whole-brain model (see Materials and Methods) fitting the neuroimaging data (Fig. 3; Kringelbach et al., 2015; Deco et al., 2017b). Briefly, whole-brain models link anatomic structure with functional dynamics (Fig. 3*A*). SC data can be obtained by DWI/DTI combined with probabilistic tractography, which represents the density of fibers between brain regions. The global dynamics of the whole-brain model results from the mutual interactions of local node dynamics coupled through the underlying empirical anatomic SC matrix. Here, we use for each brain area a local dynamical model given by a normal form of a supercritical Hopf bifurcation. The normal form of a Hopf bifurcation can describe the transition from asynchronous noisy behavior to full oscillations. The main parameter that can be manipulated for fitting the empirical data and for analyzing the model is the global coupling parameter *G* (see Materials and Methods for details). The global coupling parameter *G* corresponds to the conductivity of the synaptic connections which for simplicity is considered here uniform across the brain.

We can then investigate the intrinsic ignition as a function of *G* contrasting this with the characteristics of the intrinsic ignition at the working point of the model for the optimal fit with the empirical neuroimaging data (Fig. 3*B*).

Figure 3*C* shows the quality of fitting of the empirical wakefulness data as a function of the coupling parameter *G*. We monitor two different measures, namely, (1) the correlation between the simulated and empirical static grand average FC matrices; and (2) the absolute value of the difference between the empirical and simulated averaged global levels of synchronization between the different nodes across time (Kuramoto order parameter). The fit of the static grand average FC is shown in blue as a function of the global coupling *G*, while the synchronization (mean Kuramoto order parameter) is shown in black. As can be seen, the static FC is not the best measure for constraining the model after values of around 0.1. Instead, we use the minimum of the synchronization for this purpose, since this is much better at catching the spatiotemporal behavior of the system, as we have shown elsewhere (Kringelbach et al., 2015; Deco and Kringelbach, 2016; Deco et al., 2017b, c).

Figure 3*D* shows the variability of IDMI across brain regions as a function of *G*. As can be seen from Figure 3*C*,*D*, for *G* = *0.3* at the optimal working point of the model, i.e., when the empirical data are optimal fitted with the minimum of the difference of the synchronization levels, we also find that the standard deviation across brain areas of the IDMI is maximal. This suggests that the hierarchical organization of wakefulness is maximal for the optimal working point of the model, i.e., the brain is strongly hierarchical.

**Figure 4.**
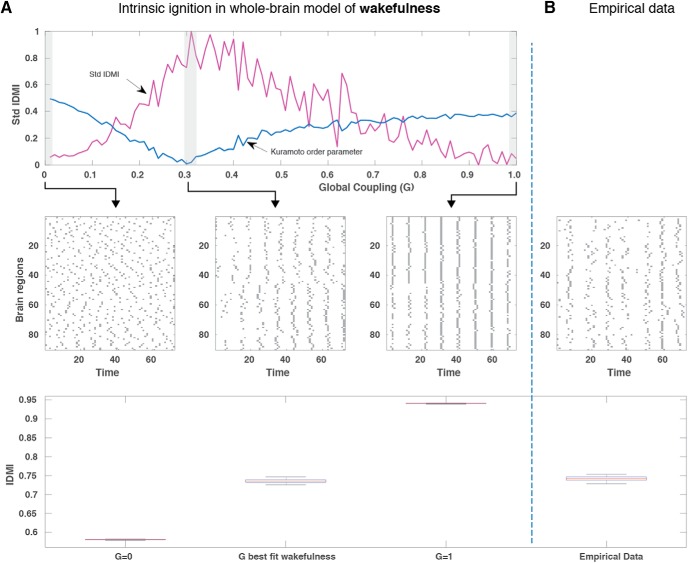
Model and ignition for wakefulness. ***A***, We show the standard deviation of intrinsic *Ignition-Driven Mean Integration* (IDMI) and the synchronization (mean Kuramoto order parameter) as a function of the global coupling point of the model fitted to wakefulness (top row). In particular, for three low, optimal, and high levels of coupling, the middle row shows plots over time of the binary intrinsic ignition events for all 90 brain regions (using the binarization process described in Fig. 1*A*). In the lower row, we show the corresponding IDMI means for these three values. ***B***, In the far-right column, this is shown directly for the empirical data with a plot over time for all brain regions (middle row) and the IDMI (bottom row).

**Figure 5.**
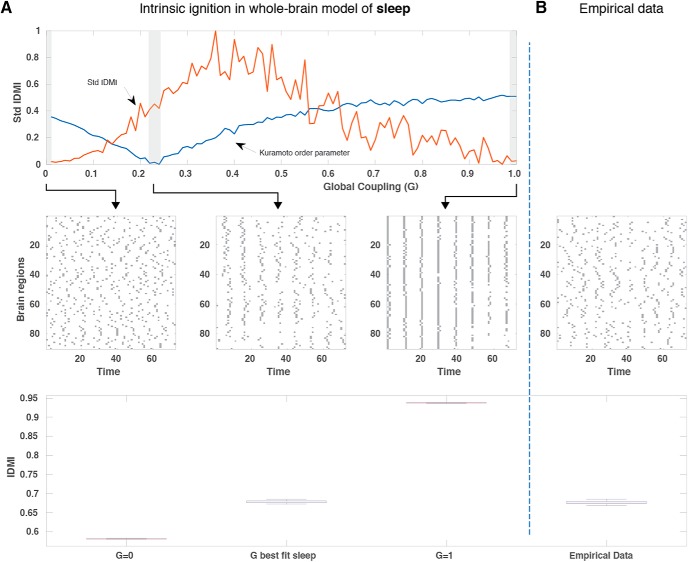
Model and ignition for deep sleep. ***A***, In the same way as in Figure 4, we show the standard deviation of intrinsic *Ignition-Driven Mean Integration* (std IDMI) and the synchronization (mean Kuramoto order parameter) as a function of the global coupling point of the model fitted to deep sleep (top row). For three low, optimal, and high levels of coupling, we show plots over time of the binary intrinsic ignition events for all 90 brain regions (middle row) and the corresponding IDMI means for these three values (lower row). As expected, the global working point of the model for deep sleep is shifted with regards to wakefulness. Similarly, at this global working point of the model, the *stdIDMI* is no longer maximal, and thus the hierarchical organization not optimal in deep sleep. ***B***, This can be compared with the plot over time for all brain regions (middle row) and the IDMI (bottom row) for the empirical data of deep sleep.

To further explore intrinsic ignition as a function of brain state, in Figures 4, 5, we fit the Hopf model for wakefulness and sleep, respectively. For the data generated by each model, we plot for three different values of *G* (small, optimal, large), the intrinsic ignition events across brain regions (over a couple of minutes) with the corresponding IDMI across brain regions. This can be contrasted with the empirical data shown on the far right. The main difference between wakefulness and sleep is that the working point shifts to a smaller value (for sleep). Importantly, for sleep the intrinsic ignition and its variability across brain regions is no longer coinciding with the optimal working point of the model.

## Discussion

In this article, we have demonstrated that the novel concept of intrinsic ignition is a very useful measure for characterizing the dynamical complexity of different brain states. Here, we have shown that the method can significantly distinguish wakefulness and deep sleep to further explore the validity of intrinsic ignition, we also used whole-brain computational modeling to causally demonstrate that the intrinsic ignition is maximal at the optimal working point. Taken together, the findings strongly suggest that this novel data-driven method can be used to fully characterize any given brain state since the intrinsic ignition is sensitive to the global coupling of the whole-brain computational model, which is regulating the dynamical complexity. Interestingly, the optimal working point of the whole-brain computational model is shifted for deep sleep which could indicate subcriticality. Future studies should use the methodology of Palva et al. (2013) to investigate the criticality of sleep compared to wakeful resting state.

The intrinsic ignition concept can provide new information on how information processing in the human brain is dependent on the hierarchical structural organization of the brain (Mesulam, 1998). It is clear that for a given brain state, this hierarchy of information processing has to allow for the optimal integration and segregation of information (Deco et al., 2015). Connectomics has made great strides in identifying the structural backbone of connectivity, identifying a rich-club containing hubs that can enable this balance (van den Heuvel and Sporns, 2011). In addition to these structural measures, it has become clear that functional measures are needed to complement our understanding of how brain regions have been demonstrated to play key functional binding roles without necessarily having the structural rich-club properties (Misic et al., 2014; Deco et al., 2017a).

Using the intrinsic ignition concept and specifically measuring the IDMI for different brain states can provide new information on the link between the structural and functional hierarchical connectivity. The present results show maximal standard deviation of the IDMI across brain regions at the working point optimally fitting the empirical wakefulness data. This shows that the functional brain organization is maximally hierarchical during spontaneous waking brain activity. This is strong evidence on the question of functional hierarchical brain organization that has been debated since the beginning of neuroscience with early physiologists like Charles Sherrington suggesting that there was a final common pathway for all brain processing, i.e., that sensory stimuli have to be processed before integration by higher order brain regions and finally executed at the top of the hierarchy in the motor cortex (Sherrington, 1906). In the vein of these observations of dynamic processing hierarchy, Baars, Dehaene, and Changeux have suggested that there could be a global workspace of brain regions controlling and broadcasting information (Baars, 1989; Dehaene et al., 1998). The global workspace regions would be at the top of the brain hierarchy whereas the sensory regions are lower down. In terms of a ranking of the intrinsic ignition, one would expect a clear staircase profile reflecting the presumed increase in computational load from sensory regions to regions in the global neuronal workspace.

Ranking the intrinsic ignition of wakefulness (and of deep sleep) shows an inverse sigmoidal curve. This demonstrates that the functional organization of brain activity is hierarchical but nonuniform graded. As such, it does not show a clear demarcation between potential workspace regions and other brain regions as predicted by the global workspace theory. Still, the results are compatible with this account, given there are clearly regions with higher intrinsic ignition variability. These are more computationally relevant and could play a central role in broadcasting information, more so than the regions with low intrinsic ignition, which are more likely to be related to sensory processing.

We also used the intrinsic ignition method to investigate differences between brain states and in particular differences between wakefulness and deep sleep. This is important given that deep sleep is generated within the same underlying anatomic structure and is a fully reversible state characterized by unresponsiveness and altered consciousness, distinguished from wakefulness by a decrease in the ability to react to stimuli (Cirelli and Tononi, 2008). Previous attempts to use ICA or seed-based methods that have been successfully used to characterize resting state data in wakefulness (Biswal et al., 1995; Greicius et al., 2003; Fransson, 2005; Fox and Raichle, 2007) have found that the same resting-state networks are generally preserved during sleep, even during deep sleep (Boly et al., 2012; Tagliazucchi et al., 2013). But it has been shown that increasing sleep depth is associated with a breakdown of corticocortical FC, accompanied by changes in brain activity (Kaufmann et al., 2006; Horovitz et al., 2009; Tagliazucchi et al., 2012b). The functional repertoire of brain connectivity is sustained by the underlying anatomic backbone (Greicius et al., 2009). Indeed, the average FC is more correlated to the underlying anatomic skeleton during states of deep sleep and anesthesia compared to wakefulness (Barttfeld et al., 2015; Tagliazucchi et al., 2016).

We found using the novel intrinsic ignition method that it significantly distinguished wakefulness and deep sleep. In fact, the IDMI in wakefulness and deep sleep were clearly separated for all of the 90 AAL brain regions across all participants (Fig. 2*C*). This demonstrates that the intrinsic ignition method is highly sensitive to different brain states.

It should be noted that while the current method uses a simple threshold method for extracting events, it could equally well use other more sophisticated mathematical methods for extracting point processes (Caballero Gaudes et al., 2013; Karahanoğlu et al., 2013; Petridou et al., 2013). Such methods have been shown to be able to describe many important aspects of dynamics such as, e.g., resting state networks and complexity (Karahanoğlu et al., 2013; Karahanoğlu and Van De Ville, 2015).

Overall, we have shown that this intrinsic ignition method is a promising method for characterizing the dynamical complexity of brain states and general principles of brain processing. In future research, it will be important to test this concept on a whole range of different brain states. One possibility would be to test conditions with changes in consciousness such as vegetative coma, minimal conscious state, locked-in syndrome and various levels of anesthesia (Casali et al., 2013). Another possibility would be to characterize altered brain states elicited by drugs such as morphine, amphetamines, psilocybin, and LSD (Carhart-Harris et al., 2014). It might also be useful for characterizing the preictal state in epilepsy to discover potential treatment targets.

Even more generally, it will be possible to use the intrinsic ignition method for detecting differences in neuropsychiatric disorders (Deco and Kringelbach, 2014). Used in conjunction with causal whole-brain computational modeling (Cabral et al., 2014), this raises the possibility to find the brain regions that lead to imbalances in the dynamical complexity associated with neuropsychiatric disorders and which could potentially be rebalanced (Kringelbach et al., 2010, 2011).

## Synthesis

Reviewing Editor: Satu Palva, University of Helsinki Decisions are customarily a result of the Reviewing Editor and the peer reviewers coming together and discussing their recommendations until a consensus is reached. When revisions are invited, a fact-based synthesis statement explaining their decision and outlining what is needed to prepare a revision will be listed below. The following reviewer(s) agreed to reveal their identity: Geza Odor, Marcello Massimini

In summary, both reviewers found your manuscript interesting, but suggested some changes to improve it. One of the main weaknesses was the lack of clarity in explaining how the taken approach and methods answer to the question presented in the manuscript. A careful revision of the text and an improved version of the methodology is therefore necessary to convince the reviewers about the validity of the proposed approach.

More specifically, it was found difficult to understand the significance of the findings, because the manuscript did not provide a clear explanation of the physiological significance for the observation that IDMIs are well separated between wakefulness and sleep but stay almost constant for any of the two brain states.

Also, the explanation and the consequences of the main results, which showed an inverse and unexpected relationship between global coupling (G) and functional connective, is lacking. What would this mean for the propagation of activity and intrinsic ignitions or could this findings be driven by spurious synchronization?

For sleep, the optimal match of the model and experiment seems to be away from the maximum of the stdIDMI. It should be discussed whether this is suggestive off-criticality.

Furthermore, also some methodological issues need clarifications.

- It should justified why the Hopf bifurcation model that has a supercritical behavior and not a model with subcritical or critical behavior was used as a whole-brain model.

- The definition of intrinsic ignition event should be clarified and clear examples of ignition events (and non-events should be given).

- It should be clarified why the undirected graphs are used instead of directed graphs to reflect integration and shown whether the Results would change if directed graphs were used.

- In order to assess that stdIDMI is effectively bell shaped versus the coupling term, a bootstrap analysis (as done in Figure 2D) should be done to ensure any of these values is effectively significant.

Minor issues

- Motivation for the usage of LS equations is needed.

- Figures 3 and 4 need improved figure legends.
